# High-efficiency nuclear transformation of the microalgae *Nannochloropsis oceanica* using Tn5 Transposome for the generation of altered lipid accumulation phenotypes

**DOI:** 10.1186/s13068-019-1475-y

**Published:** 2019-06-01

**Authors:** Hector Osorio, Carol Jara, Karen Fuenzalida, Emma Rey-Jurado, Mónica Vásquez

**Affiliations:** 1 Departamento de Genética Molecular y Microbiología, Pontificia Universidad Católica de Chile, Avenida Libertador Bernardo O´Higgins 340, Santiago, Chile; 2 Departamento de Fisiología, Pontificia Universidad Católica de Chile, Avenida Libertador Bernardo O´Higgins 340, Santiago, Chile

**Keywords:** *Nannochloropsis oceanica*, Tn5 transposon, Random mutation, CMV promoter, Cytometry

## Abstract

**Background:**

One of the major problems in the production of lipids for biotechnological purposes using microalgae is maintaining a high productivity of these molecules without reducing cellular biomass. High production rates are usually obtained by cultivating microalgae under different stress conditions. However, many of these changes usually result in lower biomass productivity. Therefore, the optimization of the culture conditions and genetic modification techniques in these organisms is needed to generate robust new strains for profitable economic use.

**Results:**

In this work, we describe a new strategy for random mutation of genomic DNA in the microalgae *Nannochloropsis oceanica* by insertion of a Transposome complex Tn5. This complex contains an antibiotic-resistance cassette commanded by a CMV viral promoter that allows high efficiency of transformation and the generation of mutants. This strategy, complemented with a large-scale identification and selection system for mutants, such as flow cytometry with cell selection, allowed us to obtain clonal cultures of mutants with altered phenotypes in the accumulation of intracellular lipids. The characterization of some of these mutants uncovered new genes that are likely to be involved in the regulation of lipid synthesis, revealing possible cellular responses that influence the intracellular homeostasis of lipids.

**Conclusion:**

The strategies proposed here are easy to implement in different types of microalgae and provide a promising scenario for improving biotechnological applications.

## Background

The combustion of fossil fuels (oil) increases the emission of greenhouse gasses that contribute to climate change, which is a threat to our planet. Fossil fuels are non-renewable sources of energy, increasingly in demand and currently insufficient to cover global energy needs [1]. Notably, microorganisms are good candidates for the production of biodiesel because of their short life cycles, low cultivation costs and great scalability. Many oleaginous microorganisms such as microalgae can accumulate lipids naturally, especially triacylglycerols (TAGs), which are the main materials for biodiesel production. Therefore, these so-called biofuels have become a promising alternative energy source for the global fuel market [2–5].

Nowadays, microalgae are considered natural factories of bioactive compounds useful for different biotechnological applications. Microalgae have been studied due to their capacity to store and produce lipids. The lipid content of *Chlamydomonas*, *Porphyridium*, *Dunaliella*, *Isochrysis*, *Tetraselmis*, *Phaeodactylum*, *Nannochloropsis*, *Chlorella* and *Schizochytrium* species varies between 20 and 50% of dry weight. Importantly, species of the *Nannochloropsis* genus are considered industrial microalgae because they produce higher amounts of lipids ranging from 37 to 60% of dry weight [4–6]. *Nannochloropsis* is a genus of unicellular photosynthetic microalgae of the class Eustigmatophyceae, ranging in size from 2 to 5 µm and widely distributed in marine, fresh and brackish water. Despite their small genomes (25.38 to 32.07 Mb), *Nannochloropsis* species have high coding potential (between 9000 to 11,000 protein-coding genes depending on the species), with many genes encoding proteins involved in the synthesis of lipids and gene redundancy for certain enzymatic steps of these biosynthetic pathways [7].

Microalgae engineering is one of the fastest growing biotechnology fields. Thus, techniques for the overexpression, suppression and editing of genes have been used in several microalgae [4, 8–11]. The sequencing and analysis of the different microalgae genomes have led to the discovery of the function of the genes involved in lipid biosynthesis [7, 12–14]. However, there are still numerous genes without any attributed function and the regulatory networks that control lipid homeostasis are largely unknown.

Different experimental approaches have been carried out to find the key genes involved in the regulation of lipid synthesis in several *Nannochloropsis* species. For example, the overexpression of enzymes involved in the early phases of lipid synthesis leads to the accumulation of lipid precursors such as acetyl-CoA and malonyl-CoA, which triggers an increase in the total production of lipids [4, 15]. Furthermore, enzymes involved in the final stages of lipid synthesis such as fatty acid desaturases and elongases [4, 15–20] have also been overexpressed. Another approach to find genes involved in lipid synthesis has been the inactivation or repression of genes by homologous recombination and CRISPR/Cas [21–24]. However, these approaches have shown to be unsuccessful in generating lipid-producing strains that can be used in biotechnological processes; they have little applicability in other microalgae, low transformation efficiency on silencing of the inserted material, and slow and laborious selection methods [25].

Due to the lack of efficient genome-editing tools for generating industrial relevant strains and the inability to increase lipid productivity without decreasing growth rate, we believe that random mutagenesis strategies may allow the identification of novel regulatory genes involved in lipid synthesis pathways. Microalgae that have been randomly mutagenized with these methods include *Isochrysis affinis galbana* [26], *Nannochloropsis* sp. [27], *Chlamydomonas reinhardtii* [28], *Pavlova lutheri* [29], *Scenedesmus dimorphus* [30], *Chorella sorokiniana* and *Scenedesmus obliquus* [31], and *Parietochloris incisa* [32]. Among the random mutagenesis strategies that have been reported, the use of Tn5 systems has many advantages compared to other genetic modification systems [32–35]. First, Tn5 is a bacterial genetic element that transposes via a cut and paste mechanism, being a powerful tool for genetic analyses [32, 33]. Second, Tn5 mutagenesis strategies are technically quite simple, since only the transposase enzyme, the transposon and the target DNA are required [34, 35]. Third, the technology is quite flexible in terms of the DNA inserted within the body of the transposon (between the two inverted repeated sequences) [34].

Although there are a variety of transformation methods that have been described for different microalgae, none of them achieve high enough efficiency to produce a large number of mutant clones to cover the entire genome. Such limited efficiency is due to several reasons: (1) the physical barriers (such as cell wall and cell membranes) that are difficult to disrupt by classic methods of transformation; (2) the transgene silencing phenomenon described in some microalgae such as *Chlamydomonas*, which precludes the expression of heterologous transgenes; (3) the laborious selection systems; and 4) the small mass of mutant cells that causes a slow identification of altered phenotypes [36–38]. In the *Nannochloropsis* model, one of the biggest problems for the insertion of foreign genetic material is the extremely rigid cellular envelope with an internal wall composed mainly of cellulose and an external layer of algaenan of the microalgae [39]. Therefore, different transformation protocols with usually low transformation efficiencies have been described. Electrotransformation, using high electric field strength, is one of the most efficient methods used for the transformation of *Nannochloropsis* [36].

In this study, we describe a novel high-efficiency method for the generation of random mutant strains at the level of genomic DNA for *Nannochloropsis* species with altered lipid phenotypes. The method includes: (1) the improvement of foreign DNA insertion into genomic DNA through the use of an in vitro Tn5 transposition complex named Transposome (transposon plus transposase), (2) the use of exogenous viral promoters for high expression of the antibiotic-resistance cassette; (3) the selection of mutant altered lipid phenotypes using flow cytometry with cell selection (FACS).

## Results and discussion

### Transformation of *Nannochloropsis oceanica* using Tn5 Transposome

**Fig. 1:**
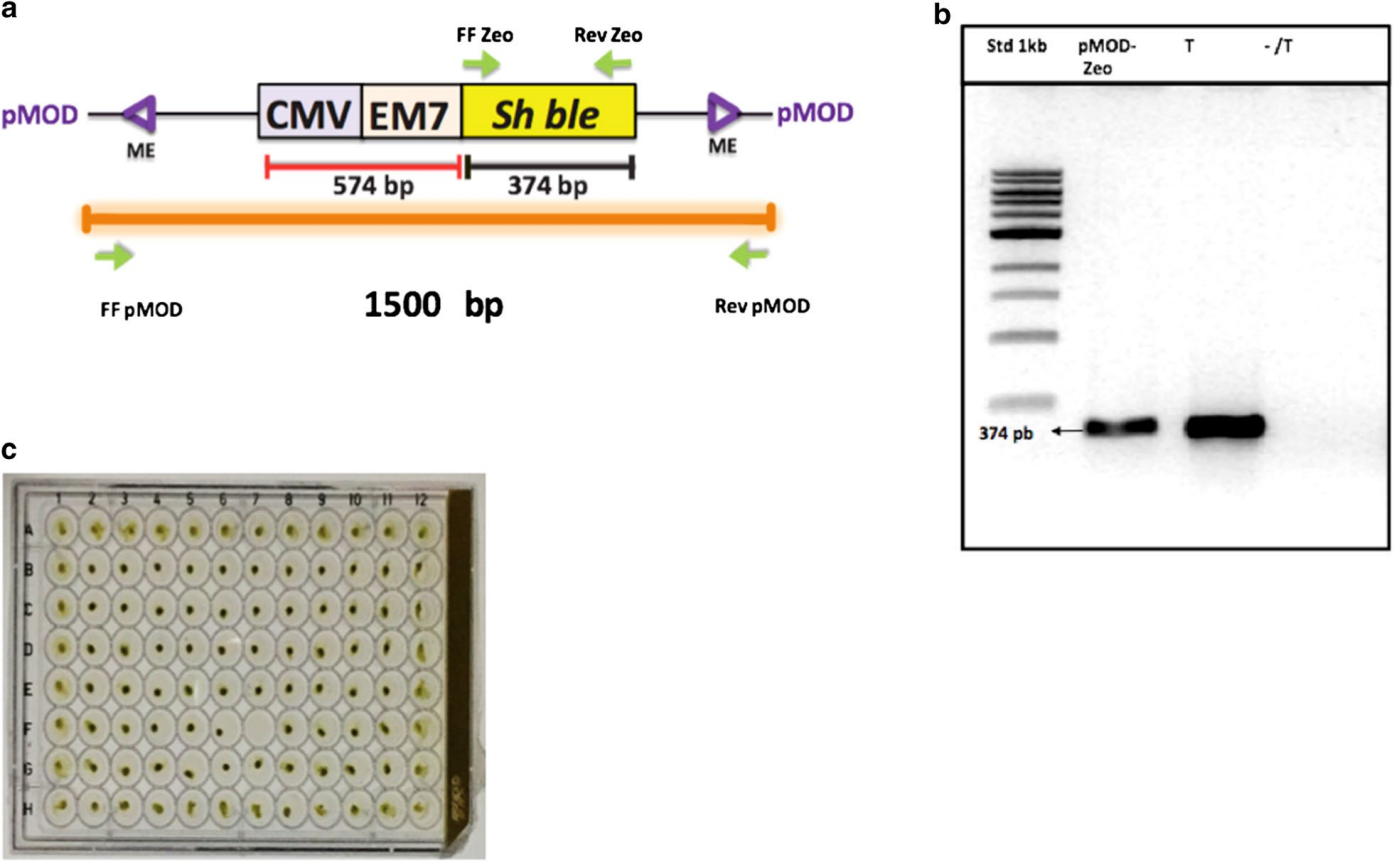
Transformation of *Nannochloropsis oceanica* using Tn5 transposome. **a** The CMV promoter allows expression of the Zeocin resistance gene in mammalian cells. The EM7 promoter is a synthetic promoter based on the bacteriophage T7 promoter for expression of the Zeocin resistance factor in *E. coli.* Sh*ble* is the gene encoding antibiotic resistance to Zeocin. Mosaic ends (ME) are recognition sites for the binding of the transposase Tn5. FF Zeo and Rev Zeo primers amplify the coding region of the Sh*ble* resistance gene. Primers FF pMOD and Rev pMOD amplify the complete construct from the pMOD vector. **b** PCR of colonies of transformed (T) and non-transformed (−/T) *N. oceanica* clones using specific primers for Zeocin resistance (374 pbs), control (+) plasmid pMOD-Zeo. C) Growth of mutant clones of transposon transformed-*Nannochloropsis oceanica* on agar plates supplemented with zeocin (2 µg/ml)

The first objective of this study was to design a molecular construct for stable and efficient transformation of the microalgae *Nannochloropsis*. To achieve this goal, we used the Tn5 transposon to favor insertion events in a random way into the *Nannochloropsis* genome. In our construct, we inserted the Sh*ble* gene between the repeated inverted sequences. This gene confers resistance to the antibiotic Zeocin, to which numerous species of microalgae present sensitivity [36, 40, 41]. The expression of the antibiotic selection gene is under the transcriptional control of two promoters: the CMV viral promoter, which has been widely described in a broad range of cell types and is the most commonly used promoter in mammalian expression plasmids [42], and the EM7 promoter, a synthetic promoter based on the bacteriophage T7 promoter for expression of the Zeocin resistance factor in *E. coli* (Fig. 1a).

The transformation of *Nannochloropsis oceanica* was performed as previously described by Kilian et al. [36] with the difference that we used the Transposome described above to be introduced into the microalgae. Using this protocol, the transformant clones were selected with Zeocin in plates and liquid cultures.

The use of the transposon resulted in transformation efficiencies (1.57 × 10^−2^ transformants per μg of DNA) much higher than those described for other types of microalgae [42–46], with a potential number of total transformants of 2,700,400 mutant clones per microgram of DNA. Importantly, nuclear transformation using endogenous promoters in *Nannochloropsis* strains has resulted in lower transformation efficiencies of around 1.25–0.6 × 10^−06^ [42, 43]. Herein, our CMV promoter/enhancer in tandem with the bacteria EM7 promoter construct design has provided up to 10,000 times higher transformation efficiency than other promoters. Thereafter, from the initial clones we isolated a total of 900 mutant clones in solid medium plates according to their lipid phenotype (HL and LL). In this work, the use of transposon Tn5, with high insertion efficiency in bacteria and fungi, also yielded high insertion rates in *Nannochloropsis,* thereby becoming a useful tool for the generation of random mutant libraries [32, 33, 36, 47].

After successive passages, the resulting cultures were expanded to a volume of 50 mL with 2 µg mL^−1^ of Zeocin. Once the genomic DNA was extracted from these mixed cultures of mutant cells, the *Shble* gene was amplified by PCR to detect the presence of the transposon (Fig. 1b). The growth of each of the clones was also tested on agar plates supplemented with the antibiotic (Fig. 1c).

### *Nannochloropsis oceanica* transformants with different altered lipid accumulation phenotypes

**Fig. 2:**
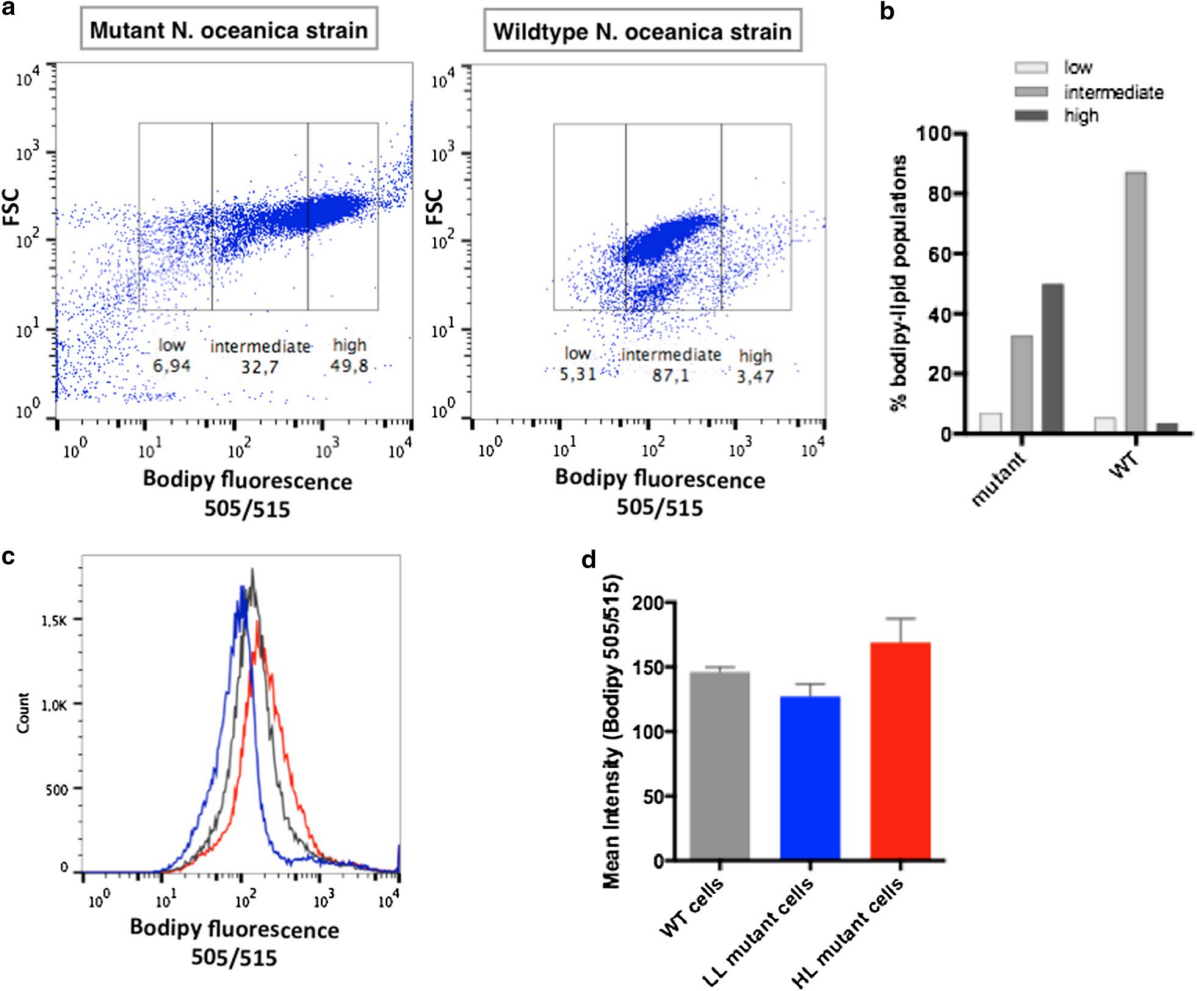
*Nannochloropsis oceanica* mutant originate heterogeneous population with different altered lipid accumulation phenotypes. Flow cytometry analysis in **a**. Dot plot of *N. oceanica* mutant and wild type strains selecting low, intermediate and high populations according Bodipy 505/515 fluorescence. **b** Fluorescence graph of Bodipy 505/515 in Low, intermediate and high populations in both mutant and wild type strains. **c** Histograms of Bodipy 505/515 fluorescence in wild type (WT), high lipid (HL) and low lipid (LL) cells in day 9 of the growth curve (stationary phase). The *Y* axis shows the number of cells and *X* axis fluorescence intensity (Bodipy 505/515). Blue curve: LL cells; Red curve: HL cells; and Gray curve: WT strain. **d** Fluorescence quantification graph of fluorophore Bodipy 505/515. The *Y* axis shows fluorescence units with their respective standard deviation (SD). All populations had the same number of cells analyzed (*n *= 50.000). Error bars in the figure represent four replicates

To study the lipid accumulation in the *Nannochloropsis oceanica (N. oceanica)* mutant strain, cells were stained with Bodipy 505/515 and processed in a flow cytometer (Fig. 2a). Each *N. oceanica* mutant strain is in fact a culture compose by a heterogeneous population consisting of several clones with altered lipid accumulation phenotypes (Fig. 2b). Analyses of the scatter-plot of FSC (cell size) and Bodipy 505/515 fluorescence (lipid-dependent, BP) showed some regions that presented these exacerbated phenotypes compared to the WT strain (Fig. 2a). Thereafter, massive analyses of mutant clones with altered lipid accumulation phenotypes were performed using the FACS technique to select cells with high (HL) and low (LL) intracellular accumulation of lipids compared to the wild type strain (WT). For each experiment, 100 cells per well were sorted from a total of 50,000 cells obtained.

Sorted HL and LL mutant cells were cultured for 9 days and then the altered lipid accumulation phenotypes were confirmed by flow cytometry (Fig. 2c). We found populations of cells with higher lipid content (HL mutants) and others with lower lipid content (LL) compared to WT cells (Fig. 2d). Importantly, fluorescent units as indicators of lipid productivity showed significant higher and lower values for HL and LL mutants, respectively, compared to WT cells (Fig. 2d). These results show the different lipid accumulation capacity of the mutant strains, phenotypes that remained stable over time.

### Sorting of single-cell lipid accumulation mutants

**Fig. 3:**
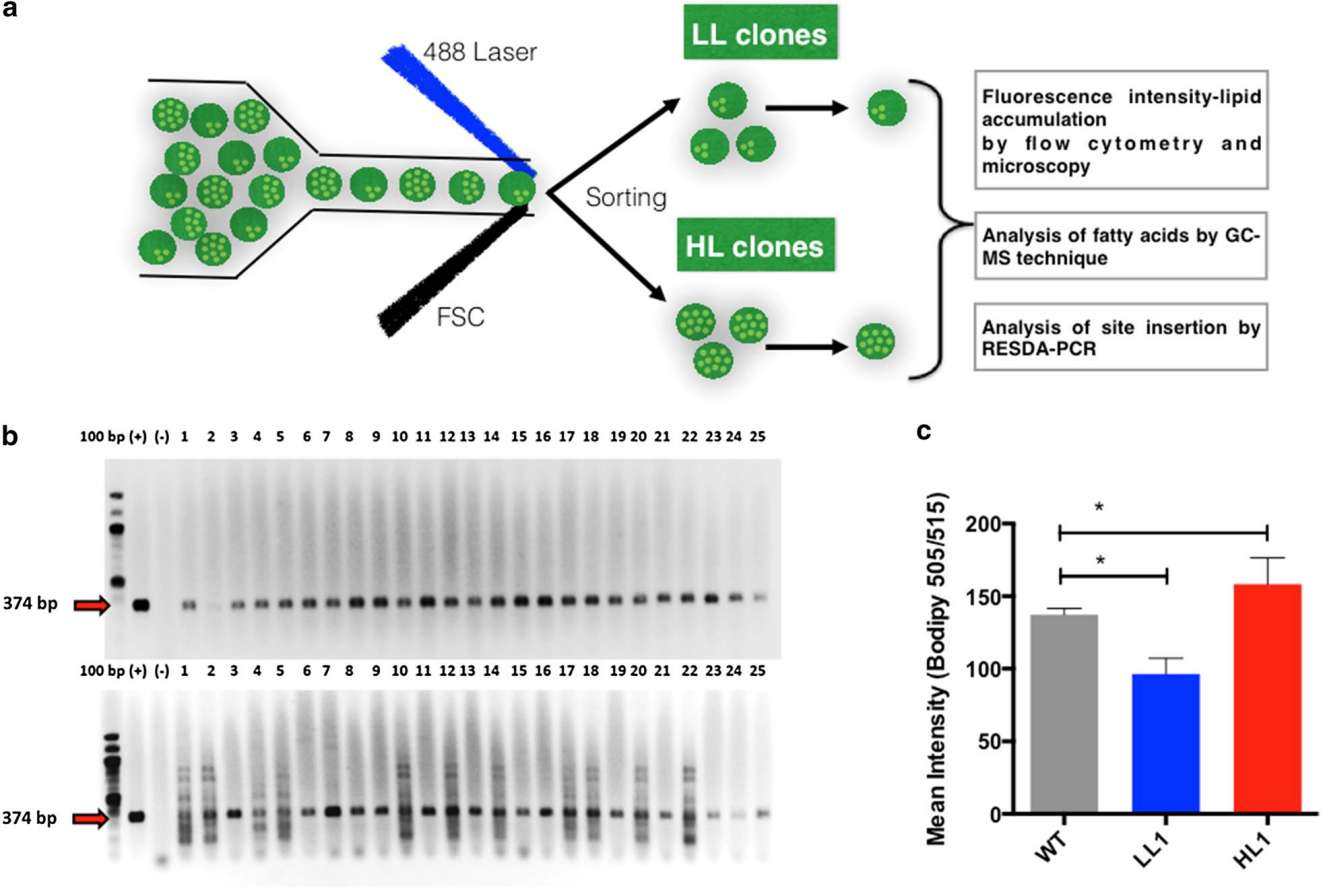
Single-cell LL and HL clones showed altered lipid accumulation. **a** Flow diagram of single-cell sorting and validation. **b** PCR validation of transposon insertion into the genome of mutant clones. The figure shows PCR products (374 bp) of the Zeocin resistance gene. (−) Negative PCR control and (+) positive PCR control with pMOD-Zeo plasmid. Also, numbers from 1 to 25 correspond to mutant clones with high-lipid phenotype (upper gel) and low lipid phenotype (lower gel). Red arrow indicates the size of 374 bp of the amplified product. **c** Validation of single-cell clones by flow cytometry, showing Bodipy 505/515 fluorescence intensity of clones. All populations had the same number of cells analyzed (*n *= 50.000) One-way ANOVA and unpaired *t* test were used (*p *< 0.05)

Because the HL and LL mutant cultures contained a mixture of clones, a new FACS selection was performed to obtain clonal cultures (1 cell per well) (Fig. 3a). After single-cell mutant selection, HL1 and LL1 clones were characterized in terms of transposon presence and lipid content.

**Fig. 4:**
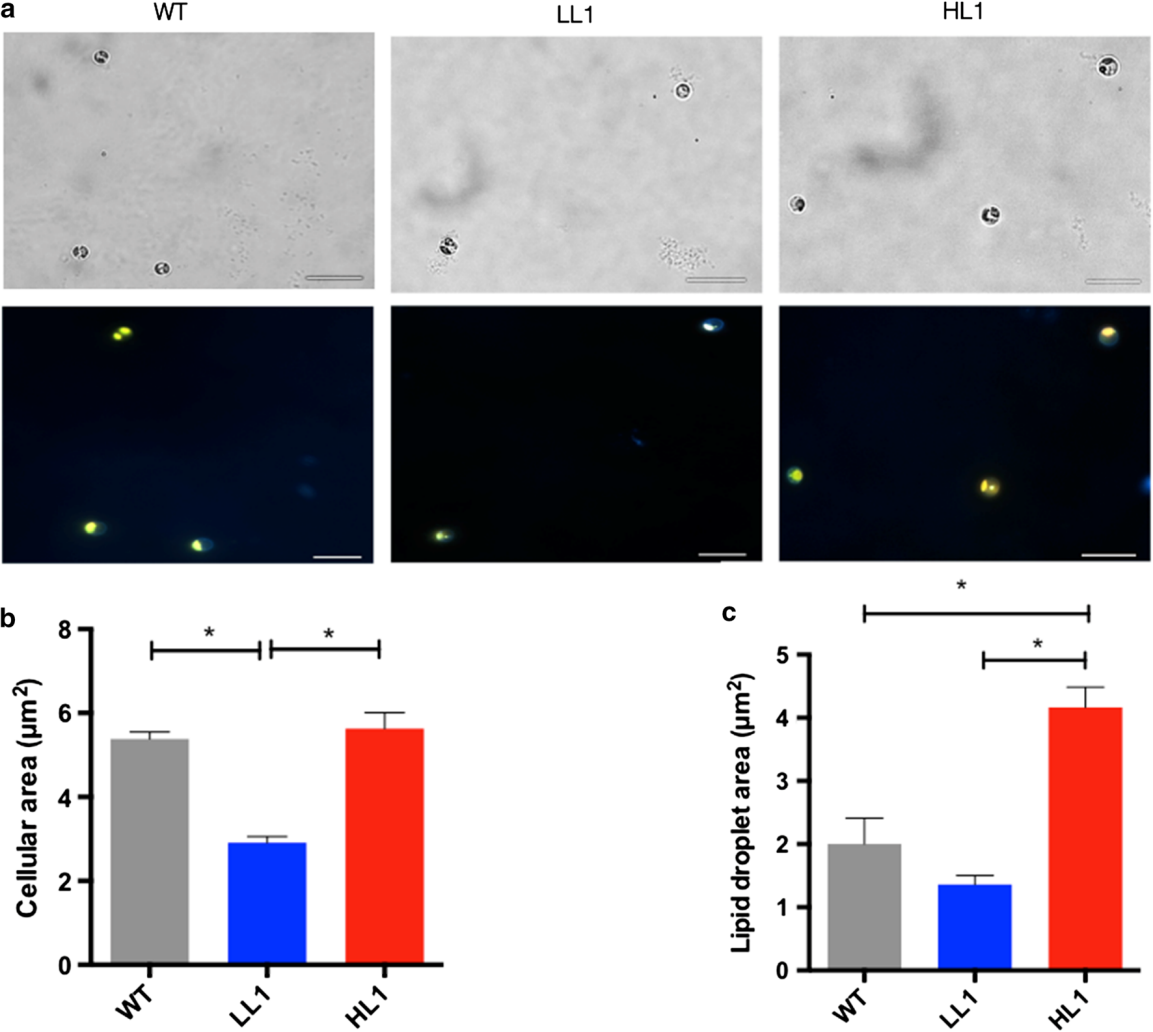
HL clone cells are bigger than WT, and LL mutant cells. **a** The images show HL1 (High-Lipid mutants), WT (Wild Type cells), LL1 (Low Lipids mutants) on bright field (upper panels) and fluorescence of lipids in green (Bodipy 505/515, lower panels) by confocal microscopy. ×100 objective magnification was used. White line indicates a size of 10 µm. **b** Cellular and **c** lipid droplet size (area) measured with ImageJ software. Error bars in the figure represent five replicates. One-way ANOVA and unpaired t test were used (*p *< 0.05)

The presence of the transposon in those single cells was confirmed by PCR, resulting positive (Fig. 3b). To verify that we obtained mutant clones of the lipid accumulation populations selected, flow cytometry was performed showing the same lipid accumulation as before single-cell sorting (Fig. 3c). Furthermore, to verify the lipid phenotype of the mutant cells, we analyzed each clone with confocal microscopy. Some differences in cell size among different cell types were found in light-field microscopy (Fig. 4a, upper panels). Consistently, HL1 and WT presented significantly larger size than the LL1 mutant (Fig. 4b). Furthermore, when examining the Bodipy 505/515 confocal images (Fig. 4a, bottom panels), we observed higher lipid content in the HL1 mutant compared with the LL1 mutant. Such observation was confirmed by quantifying the area occupied by the lipid droplets in each cell type (Fig. 4c). Such phenotypes are similar to those reported in *Chlamydomonas reinhardtii* studies using similar techniques for selecting mutant phenotypes [48].

### High-lipid and low-lipid mutant clones differ in their total oil and fatty acid profiles

**Fig. 5:**
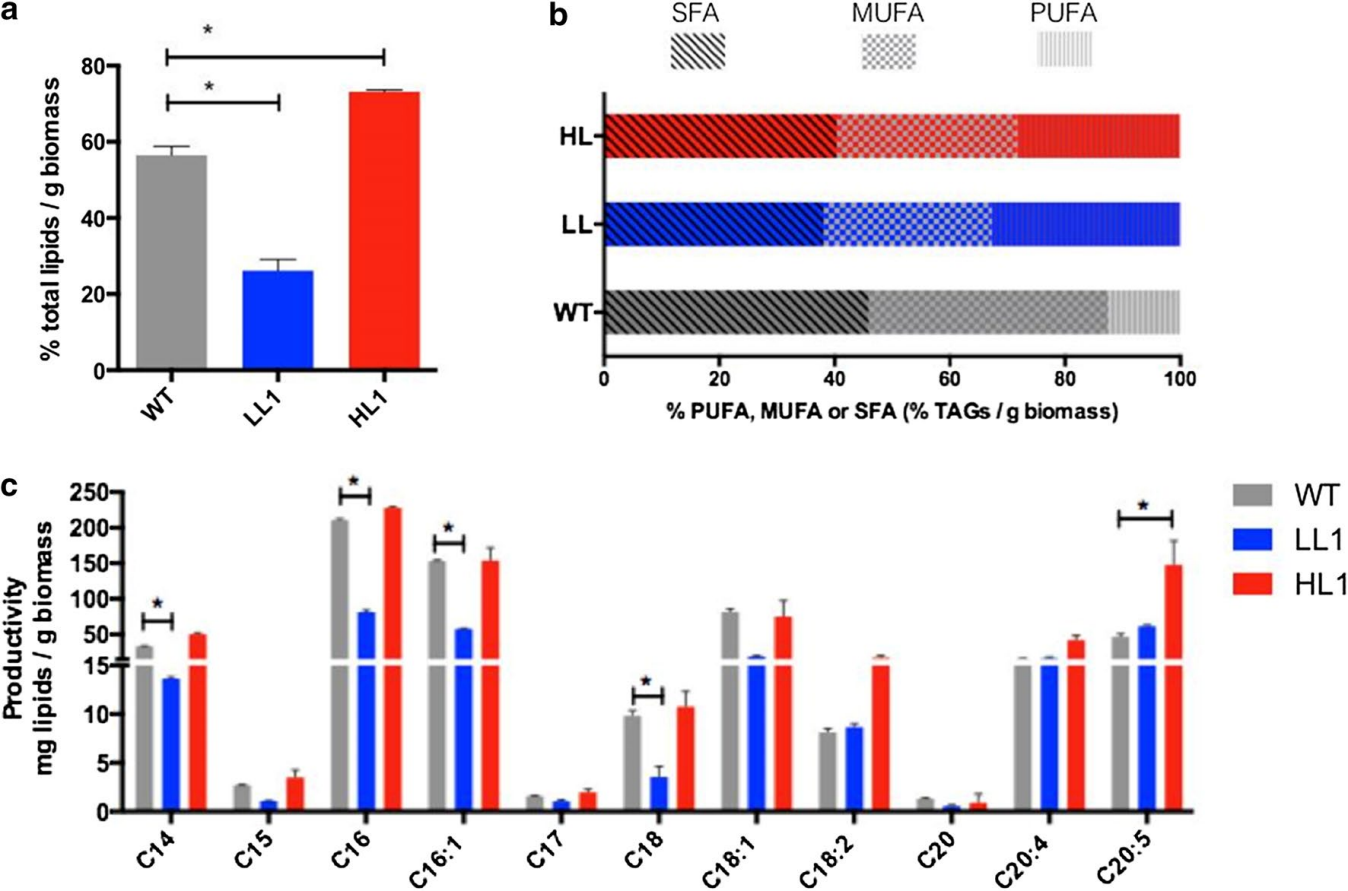
Oil and fatty acid content in wild type and mutant *Nannochloropsis oceanica* cells. Total lipids were analyzed with the Bligh and Dyer method and fatty acid profile determined by GC quantification of FAME. High-lipid cells (HL1) show a phenotype enriched in these molecules compared with the wild type (WT) and with low lipid cells (LL1). **a** Percentage of total lipids in relation to the dry weight (DW) of the microalgae. **b** The main fatty acids present in these strains. **c** Classified according to their degree of saturation (SFA, MUFA and PUFA’s as % of total TAG’s). Asterisks indicate statistical significance (2-way ANOVA, multiple t unpaired tests, *p *< 0.05)

After single-cell sorting, a high-lipid mutant clone (HL1) and a low lipid mutant clone (LL1) were chosen based on their plate growth rate and mutant phenotypes, to determine the total amount of lipids and changes in fatty acid profiles. We extracted lipids using the Bligh and Dyer technique [49] and then we performed gravimetric weighing to evaluate the total amount of lipids. The HL1 mutant clone contained high amounts of total lipids reaching 73.17% of their dry weight (Fig. 5a). These results are in agreement with what had been previously observed (Fig. 2a). Importantly, such elevated amount of intracellular lipids under normal growth conditions (without stress) has not been previously reported for this type of microalgae. In fact, it has been shown that total lipids do not exceed 60% of the cell´s dry weight in *Nannochloropsis* strains generated by interrupting internal transcriptional regulators [22] or by expressing heterologous regulators [50]. Indeed, our HL1 mutant clone exceeds the productivity of the heterotrophic industrial yeast *Yarrowia lipolytica*, which accumulates 36% and 60% of its dry weight in lipids when cultured in glucose and fed with exogenous fatty acids, respectively [51, 52]. On the other hand, the total lipid content of the LL1 mutant clone is lower than that of the WT strain, reaching 26.17% of its dry weight (Fig. 5a), consistent with flow cytometry and microscopy results.

To determine the specific changes in the fatty acid profiles of the different mutants clones, we analyzed fatty acid methyl ester (FAME) content with the GC–MS technique. As shown in Fig. 5b, the lipid profiles of the different strains are in general similar to those described in previous studies on this type of microalgae [52–55]. The most representative fatty acids in all the studied clones include myristic (C14), palmitic (C16), palmitoleic (C16:1), stearic (C18), oleic (C18:1), linoleic (C18:2), eicosatetraenoic (C20:4) and eicosapentaenoic (C20:5). Importantly, higher proportion of saturated fatty acids (SFA, 45.9%), followed by monounsaturated (MUFA, 41.7%) and polyunsaturated fatty acids (PUFAs, 12.3%) were found in the WT strain (Fig. 4c). Palmitic acid (a common component of cell membranes) is the most abundant of the saturated fatty acids. The lipid profile of the HL1 mutant showed higher content of the representative above-mentioned fatty acids, lower proportion of saturated fatty acids (SFA, 40.4%), and higher proportion of polyunsaturated fatty acids (PUFAs, 28%) compared to WT strain (Fig. 5b). Among the PUFAs, the essential fatty acid eicosapentaenoic (EPA) shows the highest increase compared with both LL and WT strains. EPA belongs to the omega-3 family of fatty acids and is commonly used as a dietary supplement for humans. Omega-3 family plays a crucial role in the prevention of cardiovascular disease, breast and colon-rectal cancer [56, 57]. Further, minor increases in the PUFAs eicosatetraenoic and linoleic acids, both belonging to the omega-6 family of fatty acids, were found in the HL1 mutant. Other fatty acids that are increased in the HL1 mutant are monounsaturated fatty acids such as palmitoleic and oleic. In contrast, lower proportion of saturated (38.1%) and monounsaturated fatty acids (29%) compared to the WT strain were found in LL1 mutant. However, as in the HL1 mutants, the proportion of PUFAs in the LL mutant clone, including linoleic (omega-6), eicosatetraenoic (omega-6) and eicosapentaenoic acid (EPA, omega-3) are increased compared to the WT (32.7%). The accumulation of PUFAs in this type of microalgae (especially EPA) has been found in response to nutritional and environmental changes such as light, low temperature and nitrogen concentration [58–61]. The increased accumulation of PUFAs found in our HL1 and LL1 mutants might be associated with regulatory changes in the synthesis of lipids or with physiological changes associated with each particular mutation. Further studies on the global cellular implications of all mutations generated in *N. oceanica* are necessary to better understand the nature of the mutants generated.

### Identification of insertion sites of the transposon in the genomic DNA of the high-lipid and low-lipid mutant clones

**Fig. 6:**
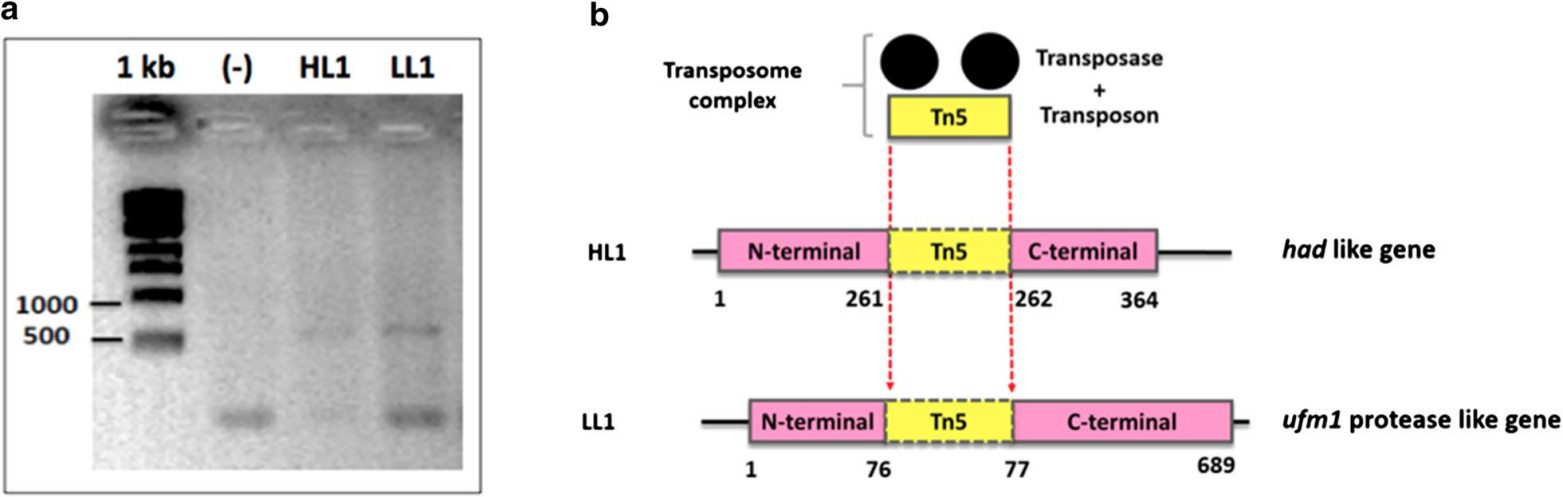
RESDA PCR amplification of the region adjacent to the transposon. **a** Results of RESDA PCR amplification for high-lipid (HL1) and low-lipid (LL1) mutant clones. 1 Kb: molecular weight standard; (−) negative PCR control. **b** Schematic representation of the transposon insertion sites into the genome of mutant cells HL1 and LL1 after comparing product PCR sequences and *N. oceanica* genome

To identify the transgene integration site within the genome, we used the RESDA-PCR technique in both selected clones (HL1 and LL1). As a result of these amplifications, it was possible to detect the presence of a single insertion of the transposon in the genomic DNA of each clone (Fig. 6a). Thereafter, deep sequencing of those PCR products was performed. The sequence was compared to the “nr” database using the Blast software. In the case of the HL1 clone, the best blast hit was against the putative haloacid dehalogenase-like hydrolase (HAD) protein of *Nannochloropsis salina* (access number TFJ87614.1), and the transposon was inserted at amino acid position 261 of the protein (Fig. 5b). In contrast, the best blast hit in the LL1 clone was against the putative Ufm1-specific protease of *Nannochloropsis gaditana* (access number EWM30439.1), the transposon was inserted into amino acid position 76 of the protein (Fig. 6b).

HADs proteins are a large family of enzymes with low similarity at full sequence level (15–30% identity), having most of them unknown biochemical or biological function. Importantly, the dephosphorylation of mannitol-1-P for use in the production of storage sugars such as laminarin and chrysolaminarin (β(1 → 3) glucose polymers) has been found in *Nannochloropsis* [62]. Similarly, a pathway involved in the storage of sugars is blocked leading to enhanced synthesis of TAGs in *Chlamydomonas reinhardtii* [63–65]. On the other hand, ubiquitin-fold modifier 1 (Ufm1) is a posttranslational modifier present in almost all eukaryotic organisms except fungi. The Ufmylation route has been related to different cellular processes such as the control of cell growth, differentiation, and endoplasmic reticulum (ER) homeostasis [66, 67]. Therefore, whether such or not this protein modification pathway is functional in *Nannochloropsis* cells, the interruption of the gene coding for these genes could explain the lipid accumulation phenotypes found. Importantly, these routes have not been previously described in classical lipid pathways.

## Conclusion

In this work, we describe a novel high-efficiency method for the generation of random mutant strains at the level of genomic DNA for *Nannochloropsis* species. The method includes: (1) the improvement of foreign DNA insertion into genomic DNA through the use of an in vitro Tn5 transposition complex named Transposome (transposon plus transposase), which has been successfully used in various eukaryotic organisms [32, 34, 68, 69]; (2) the use of exogenous viral promoters for high expression of the antibiotic-resistance cassette [42, 70, 71]; (3) the selection of lipid accumulation mutant phenotypes using flow cytometry with cell selection (FACS).

We found the highest transformation efficiency in *Nannochloropsis oceanica* cells (1.5 × 10^−2^ transformants per μg DNA), compared with those described for this type of microalgae and other microalgae models. Importantly, the generation of random mutations is a powerful strategy for the identification of indirect and non-obvious regulatory targets of cellular pathways. This powerful strategy added to the use of the Fluorescence Activated Cell Sorting (FACS) technique allows for the rapid and massive analysis of high number of cells and their separation based on the selection of phenotypes of interest. A single cell of interest can be separated from a mixture of cells in culture allowing the amplification of clonal cultures of cells with the phenotype of interest. Importantly, we were able to select altered phenotypes in lipid production by identifying mutant cells that produced high amounts of intracellular lipids using FACS technique. These cells have potential use in the energy and food industries such as the production of essential fatty acids for human intake. Furthermore, the identification of cells with low intracellular lipid content, pinpoint key genes that regulate or participate in the synthesis of these molecules that could eventually be modified for similar purposes. Interestingly, we found that the transposon was inserted in a gene coding for a putative haloacid dehalogenase-like hydrolase and putative Ufm1-specific protease in high-lipid and low-lipid clones, respectively. Thus, those insertions might be blocking those pathways, leading to the phenotypes found. Additional studies will be required to demonstrate the scope of these routes in our mutant *Nannochloropsis* strains. We believe that the approach used for our study can be applied efficiently to different types of microalgae and that it is a powerful strategy for the identification and characterization of genes of unknown function and for the generation of mutant strains with desirable commercial phenotypes. Therefore, the set of strategies described here might be extrapolated to other types of microalgae that are refractory to genetic modification, for the generation of mutant strains for basic research and commercial purposes.

## Methods

### Microalgae strain and culture conditions

*Nannochloropsis oceanica* CCAP 849/10 was maintained in artificial sea water (Sigma–Aldrich, USA) supplemented with f/2 nutrients (NaNO_3_, NaH_2_PO_4_ and micronutrients) at 25 °C [72] and continuously illuminated with 130 μmol photons m^−2^ s^−1^. Cells were growth in a 200 mL working volume in 250 mL Erlenmeyer baffled flasks with agitation (130 rpm) at 25 °C.

### Transposon construction

The region of the plasmid pCMV/Zeo (Thermo Fisher Scientific) that contains the CMV and EM7 promoters, the Sh*ble* gene (confers resistance to Zeocin) and the SV40 polyadenylation sequence (Zeo cassette Vectors Invitrogen) was amplified and cloned into the pMOD-2 plasmid (Epicenter) between the 19 bp mosaic ends (MEs) of recognition for transposase Tn5 (Epicenter). The result is the pMOD-Zeo vector, which was used for the amplification of the transposon.

### EZ-Tn5 Transposome construction

The transposon was amplified from vector pMOD-Zeo using primers FF pMOD (5′ ATTCAGGCTGCGCAACTGT 3′) and Rev pMOD (5′ GTCAGTGAGCGAGGAAGCGGAAG 3′) provided with the EZ-Tn5 pMOD-series transposon Construction Vectors (Epicenter). The complete transposon corresponds to a size of 1500 bp. PCR products were purified from the gel band using the GeneJET Gel Extraction Kit (Thermo Scientific) and concentrated in a Speed Vac until reaching a concentration of 1 µg µL^−1^.

For the production of EZ-Tn5 Transposome, 2 µL of purified transposon (concentration 1 µg µL^−1^), 4 µl EZ-Tn5 Transposase and 2 µL 100% glycerol were mixed. The mixture was incubated at room temperature for 30 min and subsequently store at –20 °C until used for electroporation experiments. 1 μL of the EZ-Tn5 Transposome mixture was used for each electroporation experiment.

### Electroporation protocol

Electroporation was performed according to published procedures [36] with some modifications. Briefly, cells were grown in liquid medium to mid-log phase (~ 1 × 10^7^ cells mL^−1^). For each electroporation, 1–2 × 10^9^ cells were harvested by centrifugation at 7000*g* at 4 °C for 10 min. Cells were washed three times with 375 mM sorbitol before resuspension in 1 mL of 375 mM sorbitol containing 1 μg PCR transposon product. Electroporation was performed using a Bio-Rad Gene Pulser Xcell Electroporation System, set at 600 Ohms, 50 μF, and 2200 V using a 2 mm cuvette, and a single 15–20 ms pulse. After the pulse, the cells were resuspended in 5 mL of artificial sea water supplemented with f/2 and allowed to recover overnight at 22 °C in low light with shaking. Cells were then harvested by centrifugation (7000*g* at 4 °C for 10 min) and resuspended in 100 mL of artificial seawater supplemented with f/2 containing 2 μg mL^−1^ Zeocin.

### Molecular analysis of transformants

Confirmation of transposon insertion into the genomic DNA of the different *Nannochloropsis* clones: genomic DNA was isolated and PCR with Zeo primers (FF Zeo 5′ ATGGCCAAGTTGACCAGTG 3′ and Re Zeo 5′ TCAGTCCTGCTCCTCGG 3′) was performed to amplify a fragment of the selectable marker gene Sh*ble*. All the resistant clones evaluated contained a DNA fragment of the correct size (374 bp) and no amplified product was obtained from WT cell lines.

### Resda-PCR

RESDA PCR was used to identify the insertion sites of the transposon in the genomic DNA as previously described [50].

This technique is based on the random distribution of frequent restriction sites in a genome and the use of degenerate primers with binding sequences to restriction sites. Specific primers of the marker DNA combined with the degenerate primers allow amplification of DNA fragments adjacent to the insertion marker using two rounds of either short or long cycling procedures [50, 73]. The PCR reactions were performed in a final volume of 30 µL using the enzyme Taq polymerase (Invitrogen). RESDA-PCR consisted of 2 stages: First amplification using a specific primer FFCMV (5′ TGGCTGACCGCCCAACG 3′) within the transposon sequence and the DegPstI (5′ CCAGTGAGCAGAGTGACGIIIIINNSCTGCAGW 3′) degenerate primer using genomic DNA from the clones transformed with the transposon as templated. The PCR conditions for first amplification were 5 min at 96 °C followed by 20 cycles of 1 min at 95 °C, 1 min at 60 °C and 3 min at 72 °C, then 10 cycles of 1 min at 95 °C, 1 min at 40 °C, 3 min at 72 °C, and a final step of 10 min at 72 °C. For the second step, PCR amplification was performed with the primers SqFF (5′ GCCAACGACTACGCACTAGCCAAC 3′) and Q0 (5′ CCAGTGAGCAGAGTGACG 3′) using 1 mL of the PCR product from the first amplification step as the template. The PCR conditions for second amplification were 5 min at 96 °C followed by 35 cycles of 1 min at 94 °C, 1 min at 60 °C, 1 min at 72 °C, and a final step of 10 min at 72 °C. Insertion sites in genomic DNA were identified by sequencing the specific PCR bands (Macrogen, Korea). Potential protein products of those sequences were predicted using the Blast software (http://www.ncbi.nlm.nih.gov).

### Bodipy 505/515 staining

Cells in log (~ 1 × 10^7^ cell mL^−1^) and stationary (~ 1 × 10^8^ cell mL^−1^) phases were diluted to a concentration of 1 × 10^6^ cells mL^−1^ and then stained for neutral lipids using Bodipy 505/515 at 0.12 μg mL^−1^ and permeabilized with DMSO 20%. After 5 min of incubation in the dark, the samples were acquired in the BD influx cytometer or observed by epifluorescence microscopy.

### Screening and sorting of mutant clones with alterations in intracellular lipid accumulation

A flow cytometer BD influx was used for the cell acquiring and cell sorting. The fluorescence reading was obtained using an excitation of 488 nm with an argon laser. The measurements were lipid-dependent fluorescence (Bodipy 505/515) and chlorophyll-dependent (autofluorescence). Measurements of 50,000 counts for sample were saved and used for further analyses.

Cell population was divided into three groups: low, middle, and high-lipid content cells, in the plot obtained based on two-dimensional dot plot (FSC and Bodipy 505/515 fluorescence). Cell sorting was carried out using cell sorting precision mode. A 70 μm nozzle and 1–100 cells per well in 96-well plates were sorted and used in the experiments. The samples mean fluorescence intensity values and images were analyzed using flowJo 6.0 software. The tubes containing sorted cells were incubated for 12 h at dark and thereafter under constant light for 2 weeks at 130 μmol photons m^−2^ s^−1^ for its growth.

### Microscopic visualization and image size quantification in microalgal cells

A drop of the sample culture (15 μL) previously stained with Bodipy 505/515 was placed on a standard rectangular microscope slide and covered with a glass coverslip. For image acquisition, a C2 confocal microscope with a 100× magnifying oil-immersion objective was used. Bodipy 505/515 was excited at 488 nm, and its emission collected from 505 to 520 nm.

Cellular and lipid droplet size (area) were measured using the ImageJ software (particle sizing function). Five cells with high intracellular lipid content (HL), low intracellular lipid content (LL) and the wild type strain (WT), at day 9 of growth were selected. For statistical analyses we used a one-way analysis of variance (ANOVA) where we compared means from each cellular phenotype using the Graphpad Prism software.

### Analysis of total lipids and fatty acids

Total lipids were extracted from 20 mg of lyophilized biomass with chloroform–methanol solvent mixture (2:1 v/v) using a procedure similar to that described by Bligh and Dyer [49]. Fatty acid methyl esters (FAMEs) were produced from the extracted lipid by a transesterification reaction. 20 mg of lyophilized biomass were dissolved in 500 µL of distilled water and further mixed with 2 mL of hexane. The mixture was put into a screw-capped glass test tube and the mixture was heated at 100 °C for 1 h in a boiling water bath. After this procedure, the upper layer containing the methyl esters was recovered with a Pasteur pipette. 1 mL of chloroform containing 0.5 mg of heptadecanoic acid (C17:0) (Sigma-Aldrich) was added to each tube as an internal standard and finally analyzed on a gas chromatograph coupled with MS (GCMSD 7890A/5975). Analyses of lipids were performed with the MATLAB software, using the one-way analysis of variance test (ANOVA) comparing means of each cellular phenotype.

## Data Availability

Not applicable.

## Abbreviations

DNAg: genomic DNA
TAG: triacylglycerol
L.I.: intracellular lipids
N.O.: *Nannochloropsis oceanica*
kb: kilobase
bp: base pairs
WT: wild type
HL: high lipids
LL: low lipids
